# A Retrospective Study of Patient-Reported Data of Bullous Pemphigoid and Mucous Membrane Pemphigoid From a US-Based Registry

**DOI:** 10.3389/fimmu.2019.02219

**Published:** 2019-09-20

**Authors:** Janet Lee, Kristina Seiffert-Sinha, Kristopher Attwood, Animesh A. Sinha

**Affiliations:** ^1^Department of Dermatology, Jacobs School of Medicine and Biomedical Sciences, University at Buffalo, Buffalo, NY, United States; ^2^Department of Biostatistics and Bioinformatics, Roswell Park Cancer Institute, Buffalo, NY, United States

**Keywords:** bullous pemphigoid, mucous membrane pemphigoid, cicatricial pemphigoid, ocular cicatricial pemphigoid, clinical characteristics, autoimmune comorbidity

## Abstract

Bullous pemphigoid (BP) and mucous membrane pemphigoid (MMP) are rare chronic autoimmune disorders characterized by subepidermal blistering. For the United States, there is a limited amount of studies in BP and MMP that address disease demographics and clinical data. In order to more comprehensively examine disease demographics and clinical factors, we performed a retrospective analysis of patient-reported data of 138 BP and 165 MMP patients enrolled in the International Pemphigus & Pemphigoid Foundation (IPPF) disease registry from 2010–2016. Patient-reported data was compared to Physician/Investigator reported data generated in our own local patient population (Western New York; 19 BP and 43 MMP patients). We confirm a female predominance in BP (M:F ratio 1:2.1) and MMP (M:F ratio 1:4.3), and a late onset within the 6th decade of life (average age at diagnosis, 59.1 ± 17.5 years for BP and 54.8 ± 11.2 years for MMP). MMP patients were significantly more likely to have a delay in diagnosis >12 months than BP patients (38 vs. 21%, respectively). Similar to other autoimmune conditions, a large number of BP (34%) and MMP (35%) patients present with other co-existing autoimmune disorders, with the most common being thyroid disease for both groups. Increased illness activity was paralleled by an increase in severe limitations of daily activities. The vast majority of of both BP and MMP patients received high intensity immunosuppression (49%). However, the majority of BP patients reported therapy with prednisone combined with other immunosuppressants (40%), while the majority of MMP patients received immunosuppressants other than prednisone (55%). With the exception of age at diagnosis, the clinical and demographic findings from both the national and local datasets were largely consistent with each other, and support those reported in other countries.

## Introduction

Among autoimmune blistering diseases bullous Pemphigoid (BP) is the most common, with an estimated incidence of 10 cases per million population (pmp) per year in the United States (US) and between 4.5 and 14 cases/pmp per year in central Europe (1, 2). BP is characterized by the presence of IgG autoantibodies against two hemidesmosomal proteins (BP180 and BP230) located in the epithelial basement membrane zone, leading to the characteristic clinical picture of tense cutaneous blisters. It is primarily a disease of the elderly with a higher prevalence in females than males (2, 3). BP has been associated with several neuropsychiatric disorders such as Parkinson's disease, dementia, stroke, and multiple sclerosis (4, 5) and with autoimmune diseases such as diabetes mellitus and psoriasis (6–9).

Mucous membrane pemphigoid (MMP), also known as cicatricial pemphigoid (CP), is an uncommon autoimmune blistering disease that primarily affects the mucous membranes such as the oral cavity and ocular mucosa. Ocular cicatricial pemphigoid (OCP) is a delineation of MMP that includes ocular manifestations but can also affect extraocular mucous membranes and non-mucosal skin. While blisters can heal without scarring, they often do heal with scars that can lead to permanent disfigurement and complications such as dysphagia and blindness. MMP and its variants are characterized by the presence of autoantibodies to the basement membrane zone at the epidermal-subepidermal junction of mucous membranes and occasionally skin. Autoantibodies are directed against several target antigens including BP180, BP230, laminin 332, laminin 311, a6b4 integrin, and type VII collagen (10). MMP is more common in women, and usually occurs in the fifth and sixth decades of life (10). The annual incidence was estimated to be between 0.9 and 1.3 new cases/pmp in Germany and France 2 decades ago, but has seen a rise by 2 cases/pmp in the following decade (11).

The rare nature of both diseases makes the collection of epidemiologic data with a substantial number of patients a laborious and challenging process. Presently, there is a lack of larger nationwide studies in the US assessing disease characteristics of BP and MMP. While several studies have examined mortality rates of BP in the US population (3, 12–14), studies on autoimmune comorbidities have mainly come from Europe (15) and Asia (1, 6, 8, 9). The exception is a recent large-scale cross-sectional study by Ren et al. that examined comorbitidies (not specifically autoimmune) based on discharge diagnosis codes in hospitalized patients with either a primary or secondary diagnosis of BP across the US (16). However, there was potential for misclassification of diagnosis codes for comorbidities, and clinical characteristics such as the disease severity for patients with either a primary or secondary diagnosis of BP were not assessed. To our knowledge, there have not been any reported national cohort studies performed to assess the clinical characteristics of MMP in the US.

This study presents the first nationwide analysis in the US to evaluate the clinical characteristics of BP and MMP and it's variants through a patient-reported registry established by the International Pemphigus & Pemphigoid Foundation (IPPF). We also compared the data obtained from the IPPF patient registry with data collected in conjunction with sample procurement for our local autoimmune bullous disease biorepository in which the data entry was curated by trained medical professionals. With the exception of age of onset, which was significantly lower than expected in the population participating the IPPF registry, our findings largely confirm those of previous epidemiologic studies in BP and MMP conducted in other countries including lesion location and medications. Additionally, our study reveals previously unknown information regarding delay in diagnosis, autoimmune comorbidity, and correlation between disease activity and limitations in daily activities.

## Materials and Methods

### Study Population and Data Collection

Patients diagnosed with BP or MMP that enrolled in a patient registry hosted by the IPPF between 4/14/2010 and 6/8/2016 were included in this study. The disease registry consisted of a 38-item self-reported survey of demographic information and disease characteristics that was available either online through the IPPF website at http://www.pemphigus.org/research/registry/ or via a mailed form if they did not have internet access (English and Spanish versions were available) (17). The survey was compliant with HIPAA (US Health Insurance Portability and Accountability Act of 1996). Data was exported from SurveyGizmo or from written input to Excel format and analyzed in a retrospective fashion.

Out of all patients responding to the IPPF registry, we included a total of 138 individuals with a diagnosis of BP and 165 individuals with a diagnosis of MMP including its variant OCP. Data from these individuals was used for constant variable analysis. In addition, both the BP and MMP groups included patients who had submitted >1 survey response to the disease registry months to years after the previous entry (*n* = 13 for BP and 11 for MMP/OCP). We included original and repeat entries for the analysis of variable disease parameters including co-existing autoimmune disorders, lesion location, disease activity, and medical care.

Since the registry contains patient-reported data and there is a potential of misclassification/re-call bias, a retrospective analysis of physician/investigator-reported data from a local biorepository-associated database of biopsy-confirmed cases of BP and MMP hosted in the corresponding author's laboratory was also carried out to corroborate the findings from the IPPF disease registry. The study was reviewed and approved by the University at Buffalo Institutional Review Board (IRB# 456887). Patients enrolled in the local biorepository were diagnosed based on standard clinical, biopsy (H&E and direct immunoflourescence) and serological parameters, including anti-BP180 and -BP230 levels and indirect immunoflourescence on salt-split skin. For comparability with the IPPF registry, data collected and analyzed in this study from the local biorepository was limited to patients enrolled between the years of 2008 and 2016. We included a total of 19 individuals with a diagnosis of BP and 43 individuals with a diagnosis of MMP. Again, there were a few patients who had provided data and samples more than once months to years after their first intake (*n* = 2 for BP and 11 for MMP/OCP). As before, repeat entry inclusion was only utilized for analyzing variable disease parameters and was excluded from analyzing constant variables such as demographic information and age at diagnosis.

### Statistical Analysis

For descriptive analyses, continuous variables are reported as means and standard deviations (SD), whereas all categorical variables are reported with percentages. All comparisons for categorical outcome variables utilized the Fisher's exact test, and the Mann-Whitney U-test was used for all continuous outcome variables. For all analyses, results were considered statistically significant at the *p* ≤ 0.05 level.

## Results

### Patients and Demographics

#### IPPF Patient Registry

BP patients had a significantly higher mean age at diagnosis than MMP patients (59.1 vs. 54.8 years, *p* = 0.001). There was no significant difference between males and females in terms of age of diagnosis in either the BP or the MMP groups (*p* = 0.21 and *p* = 0.40, respectively). A female predominance was observed in both groups, however, this was much more pronounced in the MMP group (ratio 4.3:1) than the BP group (ratio 2.1:1). The majority of both BP and MMP patients were Caucasian (85 and 90%) and from the US (81 and 88%) (Table 1).

**Table 1:** Baseline characteristics for BP and MMP for IPPF registry and local biorepository patients.

	**IPPF registry patients**		**Local biorepository patients**		**Comparison: IPPF vs. local**	
	**BP (*n* = 138)**	**MMP (*n* = 165)**	**BP (*n* = 19)**	**MMP (*n* = 43)**	**BP (*p*-value)**	**MMP (*p*-value)**
Age, mean (±SD), years	61.9 (16.6)^a^	58.0 (10.5)^b^	76.2 (13.2)	62.2 (9.4)^d^	<0.039	0.36
Age at diagnosis, mean (±SD), years	59.1 (17.5)^a^	54.8 (11.2)^b^	74.2 (14.0)	58.8 (9.9)	<0.001	0.02
Male	61.1 (18.3)	57.0 (12.7)	68.9 (11.6)	57.8 (11.2)		
Female	58.2 (17.1)	54.2 (10.8)	78.0 (14.8)	59.2 (9.4)		
Female, *n* (%)	93 (67)	134 (81)	11 (58)	30 (70)	0.444	0.141
Ethnicity, *n* (%)					0.319	0.062
Caucasian (non-Ashkenazi Jewish)	117 (85)	149 (90)	14 (74)	34 (79)		
Ashkenazi	6 (4)	2 (1)	3 (16)	4 (9)		
South Asian	2 (1)	2 (1)	1 (5)	1 (2)		
Hispanic	2 (1)	5 (3)	1 (5)	0		
African American	2 (1)	0 (0)	0	1 (2)		
Asian	5 (4)	3 (2)	0	1 (2)		
Other	4 (3)	4 (2)	0	1 (2)		
Unknown			0	1 (2)		
Country, *n* (%)						
United States	112 (81)	145 (88)				
Other^c^	26 (19)	20 (12)				

a *7 patients had no recorded birthdate and date of diagnosis*.

b *2 patients had no recorded birthdate and date of diagnosis*.

c *Other country (n) for BP patients: Australia (2), Brazil (1), Canada (8), Congo (1), India (2), New Zealand (1), Sweden (1), UK (9), unable to be determined (1). Other country (n) for MMP/OCP patients: Argentina (1), Canada (6), Denmark (1), Greece (1), Italy (1), Norway (1), UK (9)*.

d *If a patient had multiple intakes, the average of the patient's age over all intakes was used*.

For BP patients, the delay in diagnosis from the first occurrence of lesions was <3 months for 52 patients (38%), 3–6 months in 33 patients (24%), 6–12 months in 24 patients (17%), and >12 months in 29 patients (21%). For MMP patients, 27 (16%) had a delay in diagnosis of <3 months, 41 (25%) 3–6 months, 35 (21%) 6–12 months, and 62 (38%) >12 months. BP patients had a significantly higher chance to be diagnosed early (<3 months) than MMP/OCP patients (38 vs. 16%, *p* < 0.0001), while MMP patients had a significantly higher delay in diagnosis >12 months than BP patients (38 vs. 21%, respectively, *p* = 0.002).

#### Local Biorepository

The mean age of onset was considerably higher in BP than MMP patients (74.2 vs. 58.8 years, *p* < 0.001). There was no significant difference between age at the time of diagnosis and sex for either BP or MMP groups (*p* = 0.15, *p* = 0.93, respectively). The female:male ratio for MMP patients was higher than for BP patients (2.3:1 vs. 1.4:1, respectively). The majority of patients were Caucasian from both BP (74%) and MMP (79%) groups. All patients for both groups were from the US (Table 1). The mean delay in diagnosis for the 19 BP patients was 1.1 ± 2.3 years; for the 43 MMP patients it was 2.0 ± 4.6 years (delay in diagnosis information was not available for 2 BP patients and 3 MMP patients).

The mean age as well as the mean age of diagnosis was significantly lower in the IPPF registry population when compared with the local biorepository patients, with roughly 15 years difference for the BP patients and 4 years difference for the MMP patients. There were no significant differences in terms of sex and age in the two databases (Table 1).

### Disease Characteristics

#### Co-existing Autoimmune Disorders

**Figure 1:**
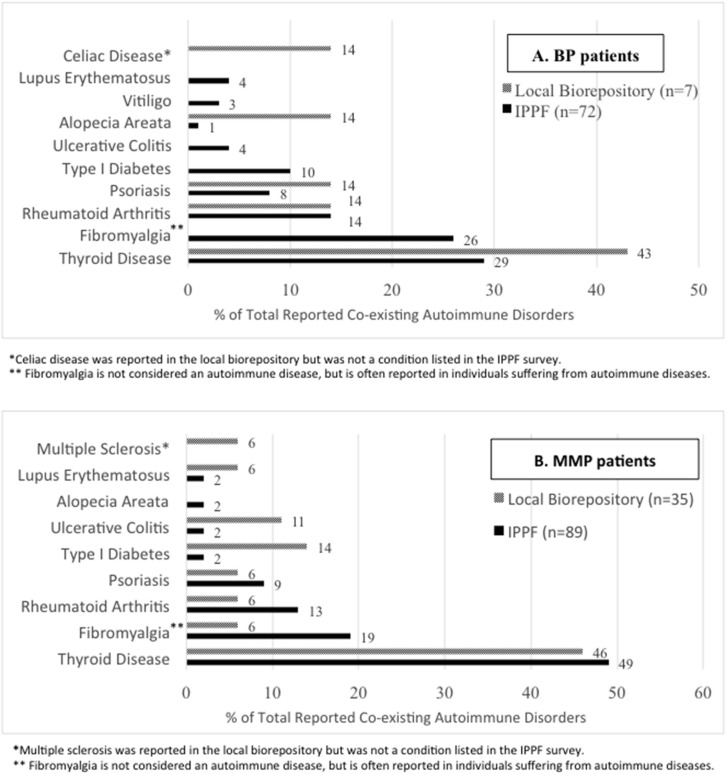
Autoimmune disease comorbidies. The percentages of specific co-existing autoimmune disorders out of all patient-reported autoimmune comorbidities are listed for the IPPF disease registry as well as the local biorepository population among **(A)** BP and **(B)** MMP patients.

In the IPPF survey, 52 (34%) of BP patients and 62 (35%) of MMP patients reported co-existing autoimmune disorder(s). Since an individual patient could have >1 co-existing autoimmune disorder, a total of 72 co-existing autoimmune disorders were recorded among BP patients, and a total of 89 co-existing autoimmune disorders were recorded among MMP patients. In the local biorepository, 5 (24%) BP patients reported 7 co-existing autoimmune disorders and 27 (50%) MMP patients reported 35 co-existing autoimmune disorder(s). The percentage of patients afflicted by at least one of the respective co-existing autoimmune diseases are presented in Figure 1. The most common co-existing autoimmune disorder reported for both BP (Figure 1A) and MMP (Figure 1B) patients was thyroid disease.

#### Lesion Location

**Figure 2:**
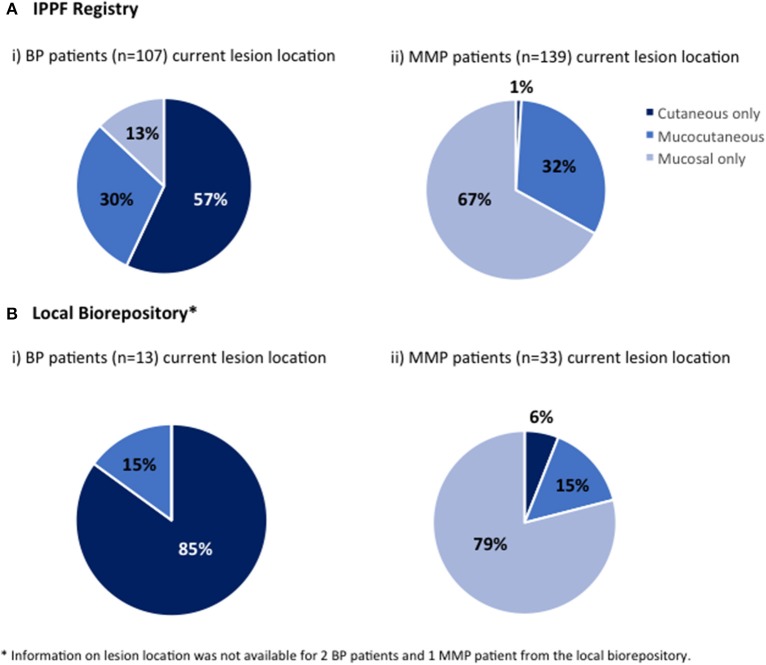
Lesion location. Patients were divided by lesion location into those who at the time of enrollment experienced mucosal lesions only, cutaneous lesions only or both mucosal and cutaneous lesions (mucocutaneous); (**A**-i) BP patients in the IPPF registry, (**A**-ii) MMP in the IPPF registry, (**B**-i) BP patients in the local biorepository, (**B**-ii) MMP patients in the local biorepository.

The location of current lesions was analyzed for both BP and MMP patients with active disease only. The majority of BP patients in both the IPPF registry and local biorepository listed their current lesion location as cutaneous only. However, the local biorepository had no patients showing mucosal only lesions with a diagnosis of BP, while 13% the IPPF registry patients reported mucosal only lesions (Figures 2A,B). The vast majority of MMP patients reported (Figure 2A) or objectively presented with (Figure 2B) mucosal only lesions. Only a small number of patients reported cutaneous only lesions. For the local dataset, we were able to confirm that the two patients that presented with cutaneous only lesions had a history of previous mucosal involvement. In order to assess what type of lesions patients can present with over the course of their disease, history of lesion location was determined for IPPF patients by combining data from both current lesions and past lesions. The self-reported results for history of lesion location in the IPPF registry were almost identical to the self-reported results for current lesions (for BP: 59% cutaneous only, 27% mucocutaneous, and 14% mucosal only; for MMP: 1% cutaneous only, 31% mucocutaneous, and 68% mucosal only).

#### Disease Activity

Patients in the IPPF survey were asked to determine their “global illness activity” defined by the following six choices: (i) no lesions and taking medication, (ii) no lesions and not taking medication, (iii) ongoing transient lesions (lasting <1 week) and taking medication, (iv) ongoing transient lesions and not taking medication, (v) repetitive lesion flares, and (vi) poor or no response to treatment. Using the guidelines established by Murrell et al. (18, 19), we defined the absence of lesions (= no lesions) as complete remission with or without therapy, ongoing transient lesions as partial remission with or without therapy, and repetitive lesion flares and poor or no response to treatment as no remission (active).

**Figure 3:**
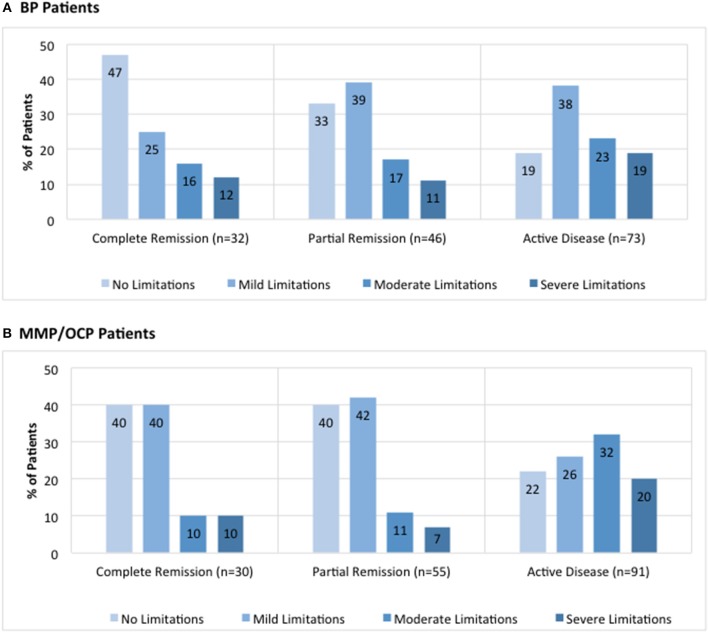
Distribution of limitations in daily activities due to lesions by global illness activity. BP patients **(A)** and MMP patients **(B)** from the IPPF registry were categorized into different groups of global illness activity consisting of either complete remission, partial remission, or active disease. For each group of global illness activity, limitations in daily activity due to lesions classified as either no limitations, mild limitations, moderate limitations, or severe limitations were analyzed. Limitation in daily activities are listed as percent limitation among all patients in a given illness activity group.

The breakdown for disease activity is presented in Figure 3 (IPPF registry BP: 21% complete remission, 30% partial remission, and 48% active disease; IPPF registry MMP: 17% complete remission, 31% partial remission, and 52% active disease).

Disease activity was also analyzed in terms of limitations in daily activities, defined by the following four choices: (i) none, (ii) mild, (iii) moderate, and (iv) severe. For BP patients, the majority of patients with complete remission reported no limitations in daily activities whereas the majority of those with active disease reported mild limitations in daily activities (Figure 3A). There was a significant difference in the number of patients that reported “no limitations in daily activities” between the complete remission and active disease groups (47 vs. 19%, *p* = 0.005). In MMP patients, the majority of patients with complete remission was split between no limitations and mild limitations in daily activities, whereas the majority of those with active disease reported moderate limitations in daily activities (Figure 3B). There was a significant difference in the number of patients that reported “moderate limitations in daily activities” between the complete remission and active disease groups for MMP patients (10 vs. 32%, *p* = 0.02).

#### Medical Care

**Figure 4:**
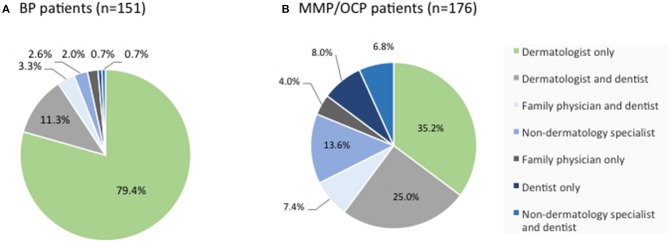
Distribution of medical provider care. BP **(A)** and MMP **(B)** patients from the IPPF registry were analyzed in terms of medical provider care. Percentages list the type of provider or combination of providers consulted for the respective skin condition out of all patients reporting medical care.

Among BP patients in the IPPF registry, the vast majority were managed by a dermatologist only, or a by a dermatologist and dentist. Only 10% of patient saw a dentist or other specialty physicians without the involvement of a dermatologist (Figure 4A). Among MMP patients, however, less than two-thirds were managed by a dermatologist only, or by a dermatologist and dentist. Forty percent of patients were managed by dentists and/or other specialty physicians without the involvement of a dermatologist (Figure 4B).

**Figure 5:**
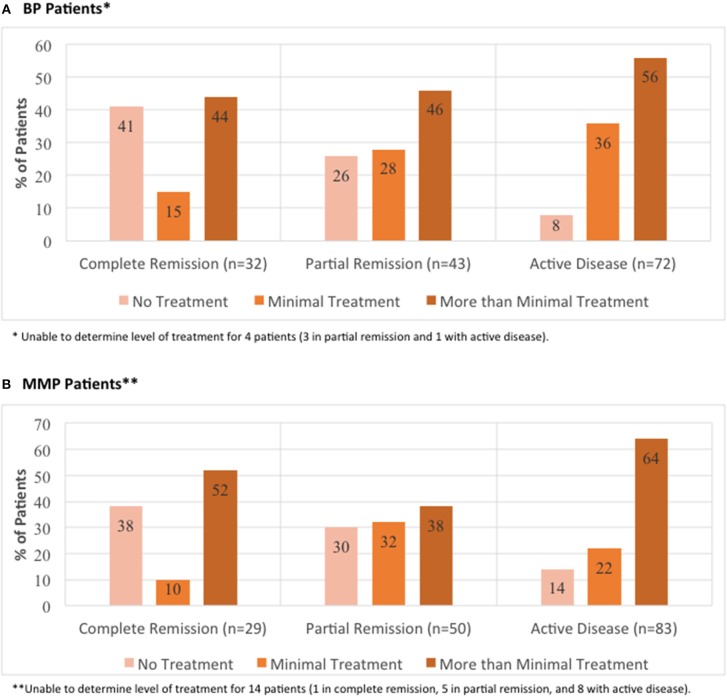
Distribution of level of treatment by global illness activity. BP patients **(A)** and MMP patients **(B)** from the IPPF registry were categorized into different groups of global illness activity consisting of either complete remission, partial remission, or active disease. For each group of global illness activity, level of treatment classified as either no treatment, minimal treatment, or more than minimal treatment was analyzed.

To compare differing levels of treatment in patient groups distinguished by levels of disease activity, therapy status was defined according to previously published consensus definitions as defined by Murrell et al. (18, 19): (i) no therapy, (ii) minimal therapy, or (iii) more than minimal therapy (anything greater than minimal therapy). Since patients did not report their weight in the IPPF survey, mean weights with respect to age, sex, and ethnicity according to the CDC were used in order to determine the level of treatment (minimal vs. greater than minimal) (20). Regardless of disease activity, the majority of patients enrolled in the IPPF repository reported being on more than minimal therapy, followed by minimal and no treatment. Similar results were obtained for patients in the local biorepository (Table 2). Amongst BP patients receiving therapy, immunosuppressive and other adjunct therapy was prescribed in the vast majority (76%) of cases, almost equally divided into a group with and without prednisone. In the MMP group, on the other hand, therapy with immunosuppressive and other adjunct agents but without prednisone was most common (55%) (Table 3). Again, the distribution of therapeutic regimens was similar in the local biorepository, with the exception of higher degrees of prednisone use in conjunction with other immunosuppressive or adjunct therapies for the BP population (Table 3). As expected, a higher percentage of patients in remission were off therapy in both the BP as well as the MMP group (41 and 38%, respectively), while only 8 or 14% of BP or MMP patients with active disease reported being off therapy (Figure 5).

**Table 2:** Level of treatment for IPPF registry and local biorepository patients.

	**IPPF registry patients**		**Local biorepository patients**	
	**BP** **(*n* = 151)**	**MMP** **(*n* = 176)**	**BP** **(*n* = 21)**	**MMP** **(*n* = 54)**
No treatment, *n* (%)	30 (20)	38 (22)	6 (29)	11 (20)
Minimal treatment, *n* (%)	43 (28)	37 (21)	4 (19)	12 (22)
More than minimal treatment, *n* (%)	74 (49)	87 (49)	10 (48)	27 (50)
Unable to be determined^a^^,^ ^b^, *n* (%)	4 (3)	14 (8)	1 (5)	4 (7)

a *Medication descriptions were not available for 3 BP patients and 13 MMP patients in the IPPF registry, and for 1 BP patient and 1 MMP patient in the local biorepository*.

b *Classification of treatment was unable to be determined for 1 BP patient and 1 MMP patient in the IPPF registry, and for 3 MMP patients in the local biorepository*.

**Table 3:** Medication use for IPPF registry and local biorepository patients.

	**IPPF registry patients**		**Local biorepository patients**	
	**BP** **(*n* = 121)^**a**^**	**MMP** **(*n* = 138)^**b**^**	**BP** **(*n* = 15)^**a**^**	**MMP** **(*n* = 43)^**b**^**
Prednisone only, *n* (%)	27 (22)	11 (8)	2 (13)	5 (12)
Prednisone + immunosuppressive agent(s) or other adjunct therapies^c^, *n* (%)	48 (40)	38 (28)	8 (53)	10 (23)
Immunosuppressive agent(s) or other adjunct therapies^d^, *n* (%)	43 (36)	76 (55)	4 (27)	27 (63)

a *Medications not available for 3 BP patients in the IPPF registry and 1 BP patient in the local biorepository*.

b *Medications not available for 13 MMP patients in the IPPF registry and 1 BP patient in the local biorepository*.

c *Immunosuppressive agent(s) or other adjunct therapies including: topical steroids, azathioprine, mycophenolate mofetil, cyclophosphamide, cyclosporine, dapsone, tetracyclines, nicotinamide, methotrexate, rituximab, etanercept, IVIg, intralesional injections*.

d *All therapies that did not include the use of prednisone, including: topical steroids, azathioprine, mycophenolate mofetil, cyclophosphamide, cyclosporine, dapsone, tetracyclines, nicotinamide, methotrexate, rituximab, etanercept, adalimumab, IVIg*.

## Discussion

There have been limited large-scale studies performed to date that have analyzed BP in the US. Although mortality rates of BP have been investigated in the US (3, 12, 14, 21), few studies have assessed comorbidities or other clinical characteristics of this disease. This study sought to improve our understanding of the clinical patterns of these chronic diseases, by using both the IPPF patient registry with large numbers of patients providing self-reported data and a local clinician-annotated biorepository and associated database for comparison.

The mean age at diagnosis for BP has been reported to range from 64 to 82.6 years in Europe, Asia, and Africa (22), whereas previous studies in the US have reported a range from 74.5 to 77 years (3, 12, 14). The data in the literature is consistent with the mean age of diagnosis of 74.2 ± 14.0 years from the local biorepository. Interestingly, the IPPF survey showed a significantly lower age of diagnosis (59.1 ± 17.5 years). Since the IPPF study was mainly collected online, the lower age at enrollment and lower age of diagnosis may be due to participation by a slighter younger, more “computer-savvy” demographic. However, both the IPPF registry and local biorepository data on sex distribution was consistent with previous studies that report a higher incidence of BP in females, with female to male ratios typically ranging from 1.04–5.1:1 (22). In MMP, the mean age of diagnosis has been described to occur mainly in the fifth or sixth decade of life (23), i.e., at a slightly younger age than in BP. Our data supports this notion, with a mean age of diagnosis for MMP patients in the 6th decade of life in both datasets. Consistent with the literature that states a female to male ratio of nearly 2:1 (10), MMP patients in both databases studied here displayed a marked female predominance, at levels almost twice of those in observed in BP.

Although patients will routinely report a delay in diagnosis before definitive diagnosis, little is known about the actual time from developing first symptoms to diagnosis for BP and MMP. Data from both the IPPF registry as well as the local biorepository indicate that, generally, BP patients have a shorter delay in diagnosis than MMP patients. Limited data in the literature support these findings with diagnosis delays in BP being on average less than a year (12), while they average more than a year for cicatricial pemphigoid (24). Not surprisingly, the period from the onset of lesions to the first hospitalization was found to be inversely correlated with body surface area (25). A possible explanation for the greater delay in diagnosis may be that patients with cutaneous lesions are more likely to seek the attention of a dermatologist, while patients with mucosal lesions only may not see a dermatologist at all or not until after diagnosis. This assumption is supported by the data on medical care summarized in this study with only 9.3% of BP patients, but 39.8% of MMP patients not being in dermatological care. Our data highlights the continuing need for educational outreach to other medical specialties.

Overall, autoimmune disease has been reported to affect 7.6–9.4% of the population (26, 27), with these individuals at an increased risk of developing a second autoimmune disease (27). In our analysis, a high prevalence of comorbidities, particularly autoimmune thyroid disease, was observed in both the IPPF survey and local biorepository for both BP and MMP patient groups. To our knowledge, few other studies in the US have assessed autoimmune comorbidities in relation to BP, and found associations with diabetes mellitus type 1 (7), SLE, celiac disease, multiple sclerosis, rheumatoid arthritis (RA) and hypothyroidism (16).

Studies from Asian countries such as Taiwan, China, Thailand, and Japan have shown some association between BP and psoriasis (6, 9), and between BP and diabetes mellitus (1, 7, 8, 28). However, European studies from the United Kingdom and Portugal found no statistical differences in autoimmune disorders in general or diabetes mellitus in particular between the BP and control groups (15, 29).

Autoimmune associations for MMP subforms are a matter of debate, showing either no additional risk of autoimmune disorders in patients with OCP (30), or demonstrating associations between OCP and RA (31), and CP with pernicious anemia (32). Our findings are in line with data on autoimmune comorbidities in Pemphigus vulgaris (33) that also showed a predominance of thyroid autoimmunity in the patient population and suggested that common genetic elements across clinically distinct autoimmune diseases may underlie autoimmune susceptibility.

In terms of lesion location, the majority of BP patients reported cutaneous only, followed by mucocutaneous, and mucosal only lesions in the IPPF patient registry, while patients in the local biorepository showed no mucosal only lesions. Prior studies from other countries have shown mucosal lesions for BP patients to either be rare with studies reporting 1.6% (28) or 5.3% (34), while others have stated higher values such as 14.3% (29), 15% (8), and 26.92% (25). For MMP patients, the majority of lesions were located on mucosal surfaces with the IPPF patient registry reporting 67% for mucosal only and the local biorepository showing 79% mucosal only.

A prior quality of life study in autoimmune bullous diseases reported that patients with mucosal involvement had a poorer quality of life than those without (35). Similarly, our findings show that a higher percentage of MMP patients with active disease reported moderate to severe limitations in daily activities compared to those with active BP. Not surprisingly, we found that increased illness activity was paralleled by an increase in moderate-to-severe limitations of daily activities. There have been a number of tools developed to assess the quality of life in patients with BP and MMP such as the Short Form Health Survey, Dermatology Life Quality Index (DLQI), and the Autoimmune Bullous Disease Quality of Life (ABQOL) questionnaire (36). However, data available regarding the usage of these tools and their outcomes is sparse. A small-scale study using the DLQI score indicated “severe impairment” of life quality, with a greater impact related to symptoms and feelings, and daily and leisure activities in BP (37). Our findings suggest that quality of life tools may be useful in clinical settings to assess disease impact.

In terms of treatment, the vast majority of patients received high intensity immunosuppression. While the majority of BP patients reported therapy with oral prednisone combined with other immunosuppressants, the majority of MMP patients received immunosuppressants other than oral prednisone. This is largely consistent with previous studies that have shown systemic corticosteroids, or a combination of systemic corticosteroids with another immunosuppressant to be the most commonly used medications in patients with BP (3, 6, 8, 29, 34, 38).

The strengths of this study include a considerable sample size that is representative of a national patient population (80% of registrants in the IPPF survey and 100% of the patients in the local biorepository were US citizens) and the collaboration between researchers, patients and a not for profit patient support organization (the IPPF). A potential limitation is the self-reporting (including the potential absence of diagnosis confirmation by a physician) and potential recall bias of patient data, particularly in regards to lesion location in the IPPF registry. Prospective studies where biopsies, salt split skin, and antibody levels are analyzed for all patients and correlated with lesion location will be useful to confirm the validity of lesional subtypes reported here. However, we compared the patient self-reported data of the IPPF patient registry to our laboratory biorepository in which data entry was curated by medical professionals in order to corroborate self-reported data and find that, with the exception of age at diagnosis, the findings from both the IPPF and our laboratory biorepository correlate well. We acknowledge that there is a risk for selection bias toward younger populations (particularly with the online format utilized for the IPPF registry), so that older patients with and without neurodegenerative diseases may not be reached. Unfortunately, comorbidities including neurologic diseases or malignancies were not assessed. In the light of newer studies showing support for an association between BP and neurologic diseases (4, 5) these conditions should be included in future questionnaires. Although the present study shows a potential association for patients with either BP or MMP with autoimmune thyroid disease, further studies need to be done to assess the validity and disease relevance of this relationship.

## Data Availability

The datasets generated for this study are available on request to the corresponding author.

## Ethics Statement

This study was carried out in accordance with the recommendations of the Institutional Review Board of the University at Buffalo for patients enrolled in the local biorepository and the Western Institutional Review board for patients enrolled in the IPPF registry with written informed consent from all subjects. All subjects gave written informed consent in accordance with the Declaration of Helsinki. The protocol was approved by the Institutional Review Board of the University at Buffalo and the Western Institutional Review board.

## Author Contributions

KS-S and AS designed the study and enrolled the local patients included in this study. JL performed data analysis. JL, KS-S and AS wrote the manuscript. KA performed statistical analyses.

### Conflict of Interest Statement

The authors declare that the research was conducted in the absence of any commercial or financial relationships that could be construed as a potential conflict of interest.
